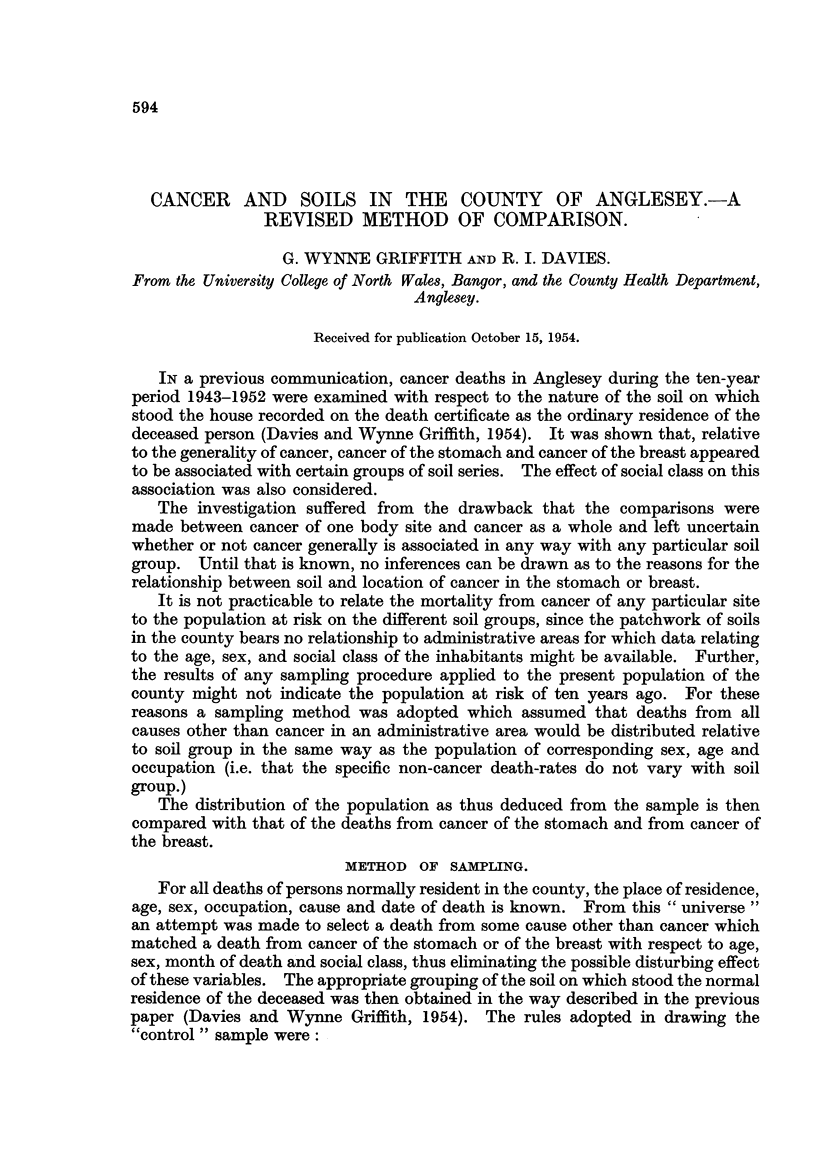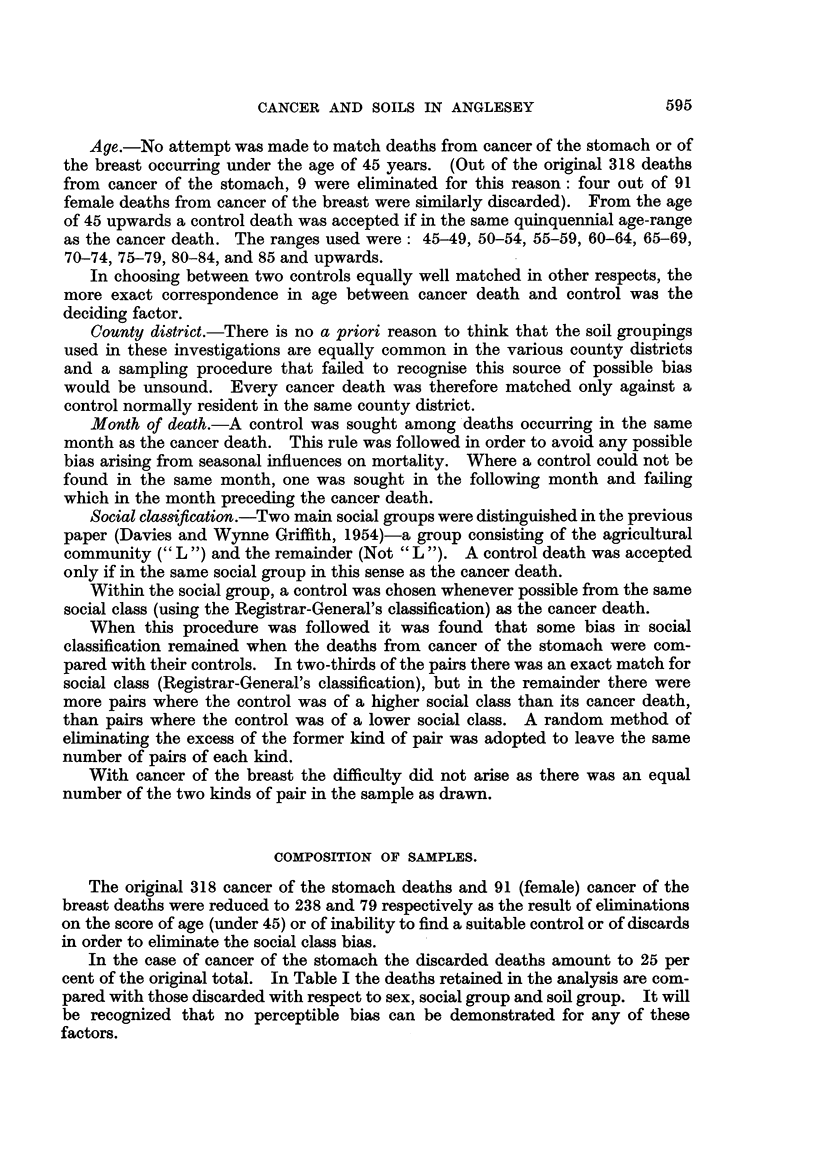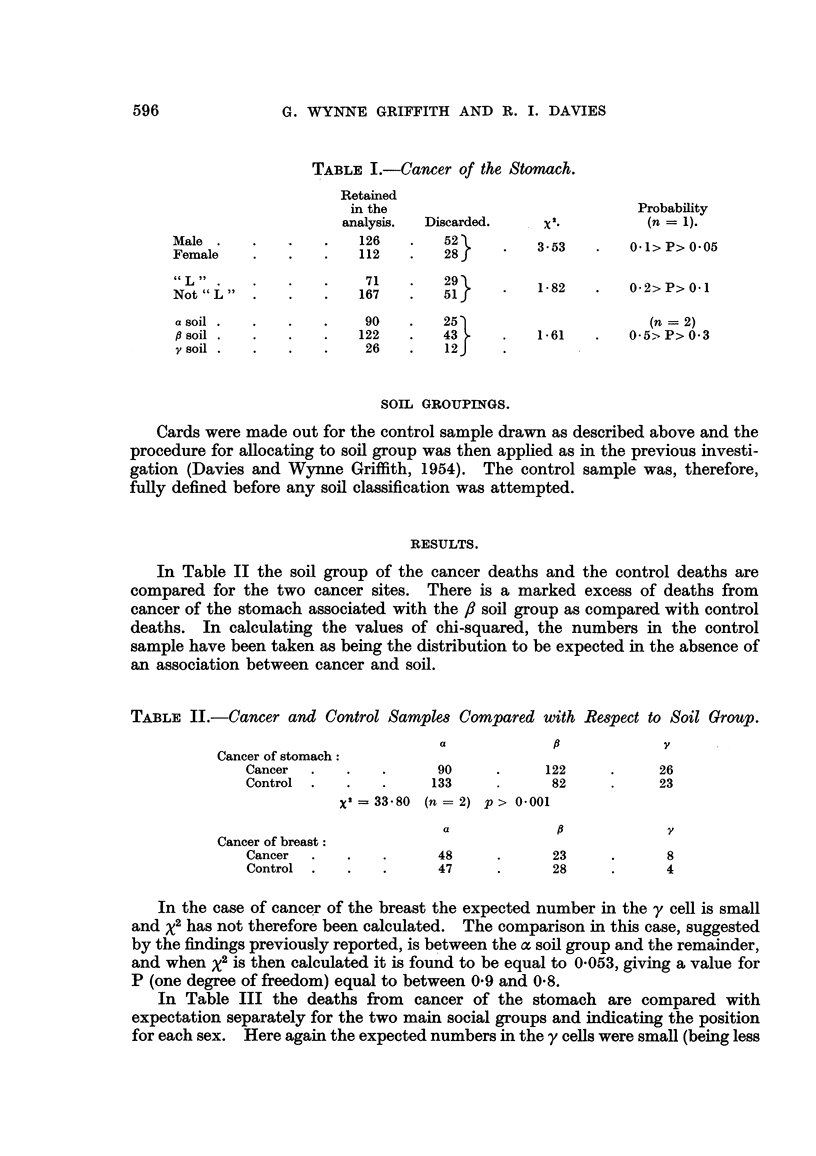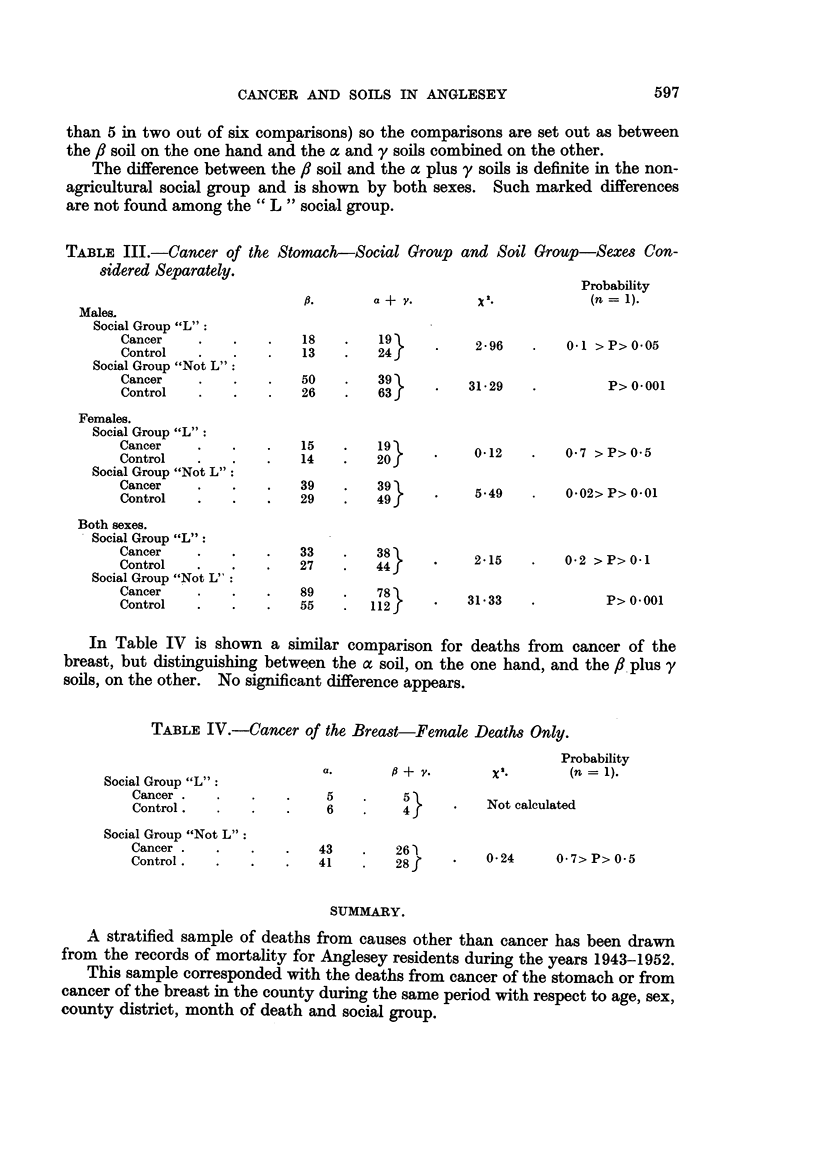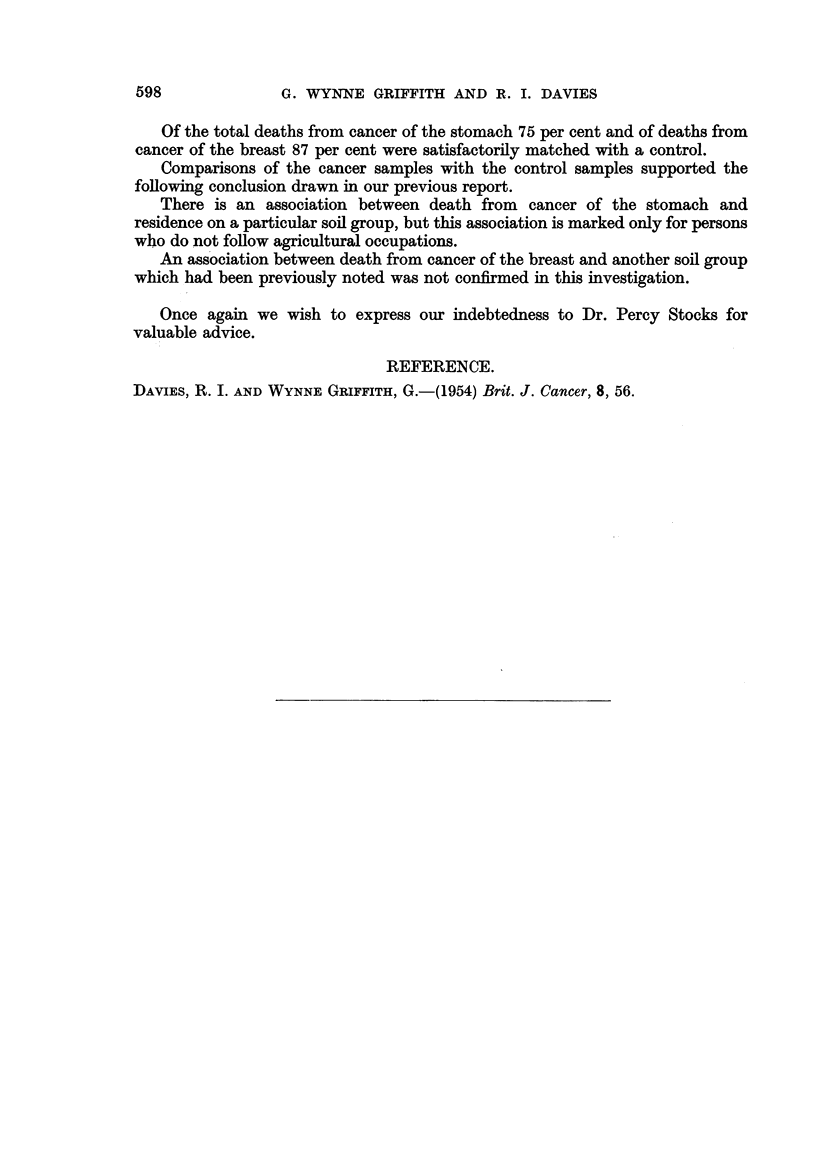# Cancer and Soils in the County of Anglesey.—A Revised Method of Comparison

**DOI:** 10.1038/bjc.1954.64

**Published:** 1954-12

**Authors:** G. Wynne Griffith, R. I. Davies


### CANCER AND SOILS IN THE COUNTY OF ANGLESEY.—A REVISED METHOD OF COMPARISON.

G. WYNNE GRIFFITH AND R. I. DAVIES.

*From the University College of North Wales, Bangor, and the County Health Department, Anglesey.*

Received for publication October 15, 1954.

In a previous communication, cancer deaths in Anglesey during the ten-year period 1943–1952 were examined with respect to the nature of the soil on which stood the house recorded on the death certificate as the ordinary residence of the deceased person (Davies and Wynne Griffith, 1954). It was shown that, relative to the generality of cancer, cancer of the stomach and cancer of the breast appeared to be associated with certain groups of soil series. The effect of social class on this association was also considered.

The investigation suffered from the drawback that the comparisons were made between cancer of one body site and cancer as a whole and left uncertain whether or not cancer generally is associated in any way with any particular soil group. Until that is known, no inferences can be drawn as to the reasons for the relationship between soil and location of cancer in the stomach or breast.

It is not practicable to relate the mortality from cancer of any particular site to the population at risk on the different soil groups, since the patchwork of soils in the county bears no relationship to administrative areas for which data relating to the age, sex, and social class of the inhabitants might be available. Further, the results of any sampling procedure applied to the present population of the county might not indicate the population at risk of ten years ago. For these reasons a sampling method was adopted which assumed that deaths from all causes other than cancer in an administrative area would be distributed relative to soil group in the same way as the population of corresponding sex, age and occupation (i.e. that the specific non-cancer death-rates do not vary with soil group.)

The distribution of the population as thus deduced from the sample is then compared with that of the deaths from cancer of the stomach and from cancer of the breast.

### METHOD OF SAMPLING.

For all deaths of persons normally resident in the county, the place of residence, age, sex, occupation, cause and date of death is known. From this " universe " an attempt was made to select a death from some cause other than cancer which matched a death from cancer of the stomach or of the breast with respect to age, sex, month of death and social class, thus eliminating the possible disturbing effect of these variables. The appropriate grouping of the soil on which stood the normal residence of the deceased was then obtained in the way described in the previous paper (Davies and Wynne Griffith, 1954). The rules adopted in drawing the "control " sample were :

*Age.*—No attempt was made to match deaths from cancer of the stomach or of the breast occurring under the age of 45 years. (Out of the original 318 deaths from cancer of the stomach, 9 were eliminated for this reason: four out of 91 female deaths from cancer of the breast were similarly discarded). From the age of 45 upwards a control death was accepted if in the same quinquennial age-range as the cancer death. The ranges used were: 45–49, 50–54, 55–59, 60–64, 65–69, 70–74, 75–79, 80–84, and 85 and upwards.

In choosing between two controls equally well matched in other respects, the more exact correspondence in age between cancer death and control was the deciding factor.

*County district.*—There is no *a priori* reason to think that the soil groupings used in these investigations are equally common in the various county districts and a sampling procedure that failed to recognise this source of possible bias would be unsound. Every cancer death was therefore matched only against a control normally resident in the same county district.

*Month of death.*—A control was sought among deaths occurring in the same month as the cancer death. This rule was followed in order to avoid any possible bias arising from seasonal influences on mortality. Where a control could not be found in the same month, one was sought in the following month and failing which in the month preceding the cancer death.

*Social classification.*—Two main social groups were distinguished in the previous paper (Davies and Wynne Griffith, 1954)—a group consisting of the agricultural community ("L") and the remainder (Not "L"). A control death was accepted only if in the same social group in this sense as the cancer death.

Within the social group, a control was chosen whenever possible from the same social class (using the Registrar-General's classification) as the cancer death.

When this procedure was followed it was found that some bias in social classification remained when the deaths from cancer of the stomach were compared with their controls. In two-thirds of the pairs there was an exact match for social class (Registrar-General's classification), but in the remainder there were more pairs where the control was of a higher social class than its cancer death, than pairs where the control was of a lower social class. A random method of eliminating the excess of the former kind of pair was adopted to leave the same number of pairs of each kind.

With cancer of the breast the difficulty did not arise as there was an equal number of the two kinds of pair in the sample as drawn.

### COMPOSITION OF SAMPLES.

The original 318 cancer of the stomach deaths and 91 (female) cancer of the breast deaths were reduced to 238 and 79 respectively as the result of eliminations on the score of age (under 45) or of inability to find a suitable control or of discards in order to eliminate the social class bias.

In the case of cancer of the stomach the discarded deaths amount to 25 per cent of the original total. In Table I the deaths retained in the analysis are compared with those discarded with respect to sex, social group and soil group. It will be recognized that no perceptible bias can be demonstrated for any of these factors.

TABLE I.—*Cancer of the Stomach.*

| | Retained in the analysis. | Discarded. | $\chi^2$. | Probability ($n = 1$). |
|---|---|---|---|---|
| Male | 126 | 52 | 3·53 | 0·1> P> 0·05 |
| Female | 112 | 28 | | |
| "L" | 71 | 29 | 1·82 | 0·2> P> 0·1 |
| Not "L" | 167 | 51 | | |
| $\alpha$ soil | 90 | 25 | | ($n = 2$) |
| $\beta$ soil | 122 | 43 | 1·61 | 0·5> P> 0·3 |
| $\gamma$ soil | 26 | 12 | | |

SOIL GROUPINGS.

Cards were made out for the control sample drawn as described above and the procedure for allocating to soil group was then applied as in the previous investigation (Davies and Wynne Griffith, 1954). The control sample was, therefore, fully defined before any soil classification was attempted.

RESULTS.

In Table II the soil group of the cancer deaths and the control deaths are compared for the two cancer sites. There is a marked excess of deaths from cancer of the stomach associated with the $\beta$ soil group as compared with control deaths. In calculating the values of chi-squared, the numbers in the control sample have been taken as being the distribution to be expected in the absence of an association between cancer and soil.

TABLE II.—*Cancer and Control Samples Compared with Respect to Soil Group.*

| | $\alpha$ | $\beta$ | $\gamma$ |
|---|---|---|---|
| Cancer of stomach : | | | |
| Cancer | 90 | 122 | 26 |
| Control | 133 | 82 | 23 |
| $\chi^2 = 33 \cdot 80$ ($n = 2$) $p >$ 0·001 | | | |
| | $\alpha$ | $\beta$ | $\gamma$ |
| Cancer of breast : | | | |
| Cancer | 48 | 23 | 8 |
| Control | 47 | 28 | 4 |

In the case of cancer of the breast the expected number in the $\gamma$ cell is small and $\chi^2$ has not therefore been calculated. The comparison in this case, suggested by the findings previously reported, is between the $\alpha$ soil group and the remainder, and when $\chi^2$ is then calculated it is found to be equal to 0·053, giving a value for P (one degree of freedom) equal to between 0·9 and 0·8.

In Table III the deaths from cancer of the stomach are compared with expectation separately for the two main social groups and indicating the position for each sex. Here again the expected numbers in the $\gamma$ cells were small (being less than 5 in two out of six comparisons) so the comparisons are set out as between the $\beta$ soil on the one hand and the $\alpha$ and $\gamma$ soils combined on the other.

The difference between the $\beta$ soil and the $\alpha$ plus $\gamma$ soils is definite in the non-agricultural social group and is shown by both sexes. Such marked differences are not found among the " L " social group.

TABLE III.—*Cancer of the Stomach—Social Group and Soil Group—Sexes Considered Separately.*

| | $\beta$. | $\alpha + \gamma$. | $\chi^2$. | Probability ($n = 1$). |
|---|---|---|---|---|
| Males. | | | | |
| Social Group "L" : | | | | |
| Cancer | 18 | 19 | 2·96 | 0·1 > P > 0·05 |
| Control | 13 | 24 | | |
| Social Group "Not L" : | | | | |
| Cancer | 50 | 39 | 31·29 | P > 0·001 |
| Control | 26 | 63 | | |
| Females. | | | | |
| Social Group "L" : | | | | |
| Cancer | 15 | 19 | 0·12 | 0·7 > P > 0·5 |
| Control | 14 | 20 | | |
| Social Group "Not L" : | | | | |
| Cancer | 39 | 39 | 5·49 | 0·02 > P > 0·01 |
| Control | 29 | 49 | | |
| Both sexes. | | | | |
| Social Group "L" : | | | | |
| Cancer | 33 | 38 | 2·15 | 0·2 > P > 0·1 |
| Control | 27 | 44 | | |
| Social Group "Not L" : | | | | |
| Cancer | 89 | 78 | 31·33 | P > 0·001 |
| Control | 55 | 112 | | |

In Table IV is shown a similar comparison for deaths from cancer of the breast, but distinguishing between the $\alpha$ soil, on the one hand, and the $\beta$ plus $\gamma$ soils, on the other. No significant difference appears.

TABLE IV.—*Cancer of the Breast—Female Deaths Only.*

| | $\alpha$. | $\beta + \gamma$. | $\chi^2$. | Probability ($n = 1$). |
|---|---|---|---|---|
| Social Group "L" : | | | | |
| Cancer | 5 | 5 | Not calculated | |
| Control | 6 | 4 | | |
| Social Group "Not L" : | | | | |
| Cancer | 43 | 26 | 0·24 | 0·7 > P > 0·5 |
| Control | 41 | 28 | | |

### SUMMARY.

A stratified sample of deaths from causes other than cancer has been drawn from the records of mortality for Anglesey residents during the years 1943–1952.

This sample corresponded with the deaths from cancer of the stomach or from cancer of the breast in the county during the same period with respect to age, sex, county district, month of death and social group.

Of the total deaths from cancer of the stomach 75 per cent and of deaths from cancer of the breast 87 per cent were satisfactorily matched with a control.

Comparisons of the cancer samples with the control samples supported the following conclusion drawn in our previous report.

There is an association between death from cancer of the stomach and residence on a particular soil group, but this association is marked only for persons who do not follow agricultural occupations.

An association between death from cancer of the breast and another soil group which had been previously noted was not confirmed in this investigation.

Once again we wish to express our indebtedness to Dr. Percy Stocks for valuable advice.

### REFERENCE.

DAVIES, R. I. AND WYNNE GRIFFITH, G.—(1954) *Brit. J. Cancer*, **8**, 56.